# Ultrasound-based statistical shape modeling for prognosis in unstable hip dysplasia

**DOI:** 10.1186/s13089-025-00419-3

**Published:** 2025-05-30

**Authors:** E. M. van Bussel, L. van Marle, J. M. Bonsel, D. de Vrij, H. Weinans, R. Sakkers

**Affiliations:** 1 Department of Orthopedic Surgery, University Medical Center Utrecht, Heidelberglaan 100, Utrecht, 3584 CX The Netherlands; 2 Department of Orthopedic Surgery, Erasmus University Medical Center, Rotterdam, The Netherlands

**Keywords:** Ultrasound, Neonatal, DDH, Unstable, Dysplasia, Hip, SSM, Statistical shape modeling, SSM

## Abstract

**Background:**

Current methods to classify developmental dysplasia of the hip (DDH) on ultrasound (US) images, such as the Graf method, provide limited prognostic information. This study aimed to improve the prediction of the clinical course and outcome at age five of decentered hips, diagnosed on the first US made in the first months after birth, by identifying acetabular shape variants on these US images using a statistical shape model (SSM).

**Patients and Methods:**

US images of the hip were retrieved from a single-center retrospective cohort of patients with DDH Graf type D/III/IV. A SSM was created from the US images made at initial diagnosis.. The association between the identified acetabular shape variants and an unfavorable outcome (residual DDH at age five and open reduction and/or a pelvic osteotomy before age five) was established with multivariable regression models.

**Results:**

92 decentered dysplastic hips with full history could be retrieved from the database and were included. At age five, 12 patients (13%) had undergone open reduction, 13 (14%) had a pelvic osteotomy, and 32 (35%) patients showed residual DDH. Four shape variants represented 95% of the variance in acetabular shape. Mode 4 was associated with an unfavorable outcome (odds ratio (OR): 1.80 (95% CI 1.12–2.90). Mode 1 was associated with less risk on open reductions or pelvic osteotomies (OR: 0.56 (95% CI 0.33–0.96).

**Conclusions:**

A potential new method of analyzing US images for DDH using SSM established four distinct acetabular shapes on neonatal US images with unstable DDH, of which two were associated with outcomes at five years of age. This tool could serve as a basis for a better prediction of outcome and a more personalized and effective guide for treatment.

## Introduction

Developmental dysplasia of the hip (DDH) covers a broad spectrum of abnormal hip joint development and consequential insufficient acetabular coverage of the femoral head. With a 1–1.5% prevalence in newborns and an incidence of 5–13 per 1000, DDH is a common condition that can result in insufficient femoral head coverage after growth. Insufficient femoral head coverage (FHC) increases the risk of osteoarthritis at a relatively early age, whereas unstable, subluxated, or dislocated hips can inhibit acetabular growth during growth and its sequelae. (Jiménezet al. [7, 21–23]) Early diagnosis and adequate treatment, especially in these cases, are therefore essential.

The initial diagnosis of DDH in newborns is primarily based on physical examination and ultrasound (US) imaging. While physical exams have low sensitivity, correctly performed US can provide an early and reliable diagnosis. (Jiménez et al. [7]) The Graf classification, which was used from the 1980s onwards, is the most used method to classify DDH on hip US images. Although this method shows a high sensitivity for diagnosis according to the Graf classification definitions, it has a low specificity for the hip's prognosis. (Chavoshi et al. [2, 4]) For unstable, subluxated, and luxated hips (Graf types D, III, IV), the consensus is that a concentric hip should be obtained as soon as possible for optimal acetabular growth by preferably closed reduction or, if necessary, open reduction. The clinical course varies, with at least 15% of the hips requiring additional surgery due to persistent residual DDH during growth [9, 21]. However, it is unclear which decentered hips have a higher risk for inadequate development after initial closed reduction, as there are no established methods to identify high-risk hips or predict their clinical course. [20]

This paper describes a specific method for analyzing and classifying US images of unstable Graf types D, III, and IV hips. Instead of measuring alfa and beta angles or femoral head coverage (FHC), the method quantifies the shape of the acetabulum’s lineation using a Statistical Shape Model (SSM) [1, 5, 17]. An SSM enables the retrieval and analysis of more data from the same US image compared to the Graf or FHC, allowing for the identification of more subtle hip shape differences. A recent study by [3] SSM could identify shape patterns in hips with stable Graf type IIb or IIc correlated with hip development over time and identified variable US probe positioning. This method of analyzing image data from hip US images might be the next step in classifying DDH with a better correlation with outcomes [3].

This study aimed to improve the prediction of the clinical course and outcome at age five of decentered hips, diagnosed on the first US made in the first months after birth, by identifying acetabular shape variants on these US images using a statistical shape model (SSM).

We evaluated whether the shapes identified by SSM were associated with (1) the risk of open reduction, (2) the risk of open reduction or a corrective pelvic osteotomy after successful closed reduction, (3) the risk of a pelvic osteotomy or residual DDH, and (4) the collective risk of open reduction, pelvic osteotomy, or residual DDH at age 5, termed ‘unfavorable outcome.’

## Materials and methods

### Patients

For the collection of hips eligible for the study, a retrospective search was performed in the database of patients who presented with untreated Graf D and Graf III and IV between 01/2002 and 02/2020 at the Department of Orthopaedics of the University Medical Center Utrecht (UMC Utrecht). The retrospective cohort design study was reviewed by the local medical ethics committee of the UMC Utrecht(research protocol number 22/061). Due to the lack of comparable studies, no sample size or power analysis could be done.

The study population consisted of neonates with a first visit around three months of age, including (untreated) referrals from non-academic hospitals. Due to the national guidelines of regionally selected centers of expertise and treatment, no selection bias was expected. Patients with untreated DDH, type D, III, or IV according to the Graf classification and a baseline ultrasound (US) made with a linear array probe were considered eligible. Patients with comorbidities affecting the musculoskeletal system were excluded.

### Data collection

Medical records were used to check the eligibility of the patients. The baseline characteristics (age, gender, co-morbidities, affected side) and clinical course, including treatments during the first five years of age, were extracted. Two authors (EvB/LvM) chose the best image of the affected untreated hip’s initial ultrasound (US) based on the anatomical structures defined in the Graf guidelines. [8] The ilium and start of the slope of the acetabular roof were marked as key anatomical structures. One hip was randomly selected in patients with bilateral dislocated DDH and equal-quality ultrasound images of both sides. For patients with no surgical treatment, the AP radiographic image of the pelvis around the age of 5 -part of standard follow-up was used to diagnose any residual DDH. Patients were excluded if data in the medical records was missing, the US images did not show clearly identifiable anatomical landmarks, or the acetabular index on the AP radiographic image at age 5 was not assessable.

### Outcome definitions

Initial closed reduction after diagnosis was not defined as an adverse outcome at follow-up. Four outcomes were defined based on our four hypotheses. The first defined adverse outcome was initial open reduction due to failed closed reduction. The second adverse outcome was defined as either open reduction or a pelvic osteotomy within the first 5 years. The third adverse outcome was defined as a pelvic osteotomy within the first 5 years or residual DDH at age 5, with all open reductions excluded. The fourth defined adverse outcome was either an open reduction, a pelvic osteotomy within the first 5 years, or residual DDH at age 5, together referred to as an ‘unfavorable outcome.’ An acetabular index of > 21° on the pelvic AP radiographic image measured by the radiologist and treating pediatric orthopedic surgeon at age 5 was considered residual DDH. Avascular necrosis (AVN) was considered a secondary outcome. Two authors checked all acetabular indices (EvB/DdV).

### Statistical shape modeling

**Fig. 1:**
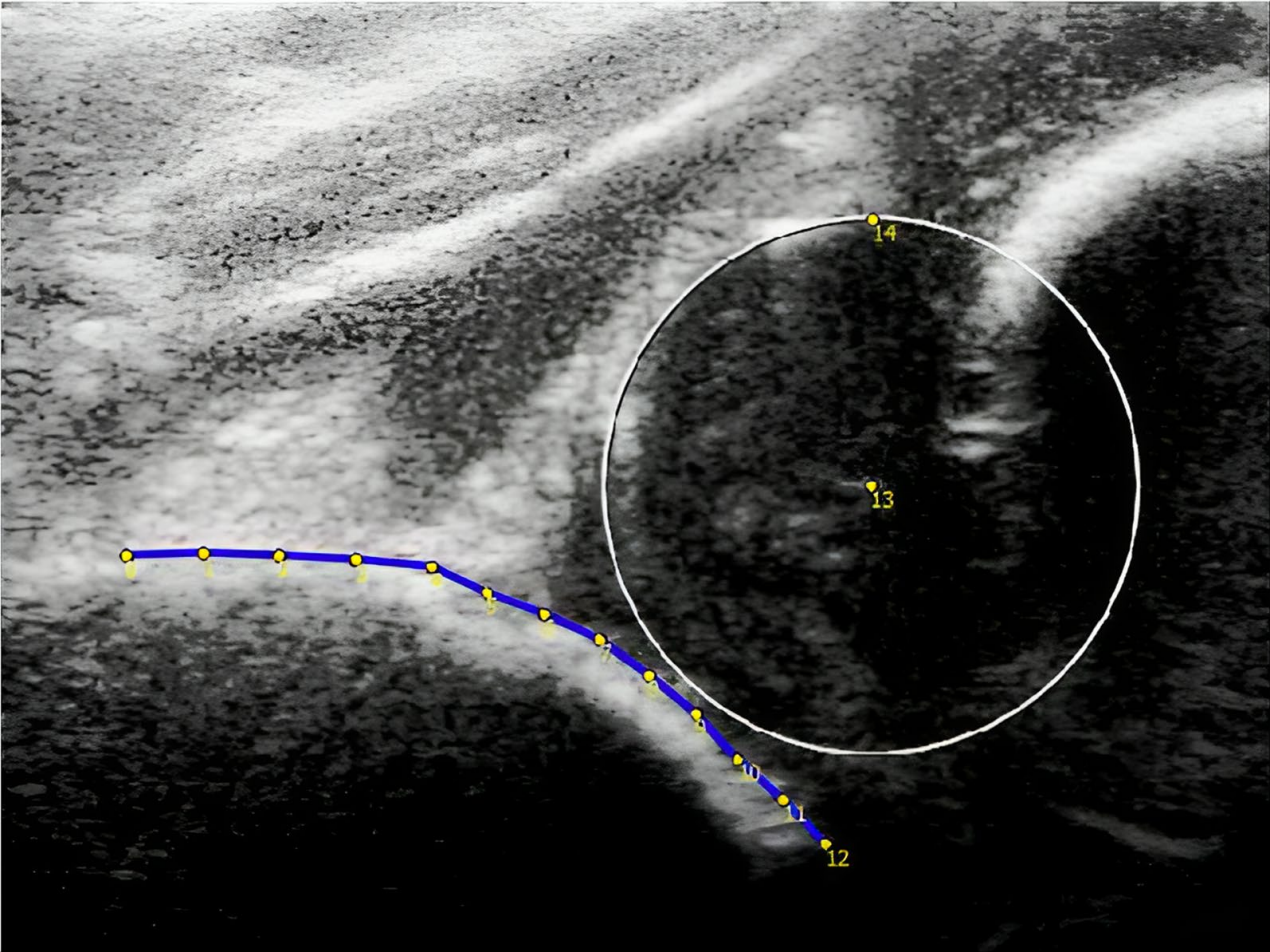
Baseline ultrasound of a decentered hip with points 0–12 at the bony contour of the ilium and acetabulum. Points 13 and 14 are placed on a best-fitted circle drawn over the femoral head

SSM was applied to the US images using BoneFinder (version 1.3.0) [14]. SSM enables quantification and analysis of subtle shape differences in images, compared to currently used less specific geometric data, such as the angles in the Graf method. The quantification of images in this SSM was based on the Statistical Shape Model used by Bonsel et al. [3] For every single US image, the shape of the acetabulum was marked using a set of thirteen manually placed predefined points, numbered 0–12 (Fig. 1). As used in the Graf method, the anatomical landmark of point 4 was defined as the start of the osseous deviation from the straight line drawn over the ilium. Point 12 was the deepest visible osseous acetabular point in the US image. Point 0 was placed at the ilium at half the distance from points 4 to 12, a slight difference compared to the method from the study by Bonsel et al., in which point 0 was placed at the image's border. This way, the shape variation due to probe positioning, represented by large variations in the ilium-to-acetabulum length ratio, was minimized. All other points were manually placed over the osseous ilium in BoneFinder– acetabular border with equal spacing using software scripts written in R^©^ (version 4.3.1) [18].

Next, the complete set of points was decomposed into several independent shape modes using principal component analysis. Within the SSM, each shape mode explains a unique aspect of shape variation within the studied population. This study retained the shape modes explaining 95% of the shape variation. In the total image set, the first shape mode explained the highest proportion of variation, which decreased with each mode. Each image has a value on each mode, expressed in a standard score, or Z-score, which describes the deviation from the mean in that shape mode. For example, a ± 1 Z-score in mode 1 would indicate that the shape of the line representing the acetabular geometry deviates ± 1 standard deviation (SD) from the mean shape in mode 1. In this study, every image of the hips was described or quantified by a set of Z-scores of the shape modes found.

Z-scores were categorized into quintiles to determine whether the association between specific Z-score values and outcomes was linear. The presence of either a linear or exponential association was evaluated by checking the odds ratios for these categories. This resulted in potential associations between clinical outcomes and mean shape modes and possible associations between clinical outcomes with either positive or negative SDs of every shape mode.

### Femoral head coverage

In addition to the 13 landmarks used for SSM, two points (13 and 14) were placed to analyze and quantify the femoral head coverage (FHC). A circle was drawn in which the labrum and bony acetabulum determined the diameter and location; point 13 was placed in the center, and point 14 was placed at the outer border of the circle to quantify the diameter of the head. (Fig. 1). To calculate the FHC, a straight line was drawn through the femoral head, starting from point 0 through point 4, located at the border of the ilium. The depth of the femoral head relative to the border of the ilium was determined. The FHC was then determined by calculating the percentual ratio of the surface of the circle beneath this line. FHC was categorized by values described by Morin (< 33%, 33–58%, > 58%) [10, 15].

### Inter and intraobserver reliability

Reliability analysis was performed by calculating an overall combined inter- and intraclass correlation coefficient (ICC). Eighteen random images (20%) were annotated twice with a 4-month interval by the same researcher (EvB) and annotated by another researcher (LvM). Z-scores derived for each shape mode of the SSM from the second annotation and the second researcher were obtained using the same method described above and compared. An overall ICC was calculated, with every mode weighted for its share of total variation. An overall ICC > 0.70 was considered sufficient. [12, 13]

### Statistical analyses

IBM SPSS Statistics 27.0 was used for statistical analysis. Patient characteristics were analyzed descriptively and noted as mean ± SD for continuous variables, and categorical variables were indicated as number (%). Odds ratios (ORs), 95% confidence intervals (95% CIs), and p-values were calculated for the association between the different shape modes and outcomes using binary logistic regression. Shape modes were entered both as continuous and categorized (quintiles) variables. Univariable and multivariable analyses were conducted; the maximum number of covariates in a single regression model was limited by population size. For this study, the limit was set to 5, according to the thumb rule 5–10% of subjects. We entered all shape modes and FHC (categorized cf. Morin) in the multivariable model; other covariates, such as age or sex, were not entered due to the set limit of covariates. The Two Way Mixed model with absolute agreement was used to calculate the mean inter- and intraclass correlation coefficient (ICC). Single measures were used for the interpretation of the interobserver coefficient. A p-value < 0.05 was considered significant. In addition, the association between either FHC or AVN and the shape modes and the association with Graf D versus Graf III/IV were analyzed.

## Results

### Patient inclusion

**Fig. 2:**
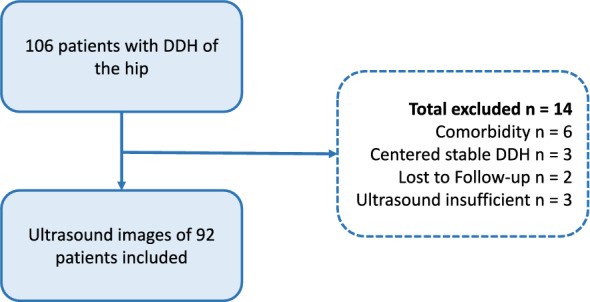
Flowchart of the inclusion process of patients

A total of 106 patients were retrieved from the database, of which 92 were included. Three patients were excluded due to missing or not assessable baseline ultrasound images. Upon re-evaluation, three hips were classified as stable, while six patients were excluded for genetic comorbidities. Two patients were lost to follow-up. Four patients had a bilateral unstable dysplastic hip. Two of these had superior imaging of one side, and two had an equal-quality ultrasound image of both sides, of which one was randomly chosen. The process of inclusion is shown in Fig. 2.

### Patient characteristics

The mean age at the baseline ultrasound was 3.4 ± SD 1.2 months. Of the 92 included patients, 77 patients (84%) were female, and 65 (71%) had the left hip included. The mean age at the defined follow-up radiograph was 5.0 ± SD 1.0 years.

Ten patients (11%) were classified as Graf type D; the other 82 hips (89%) were type Graf 3 or 4. The mean FHC index was 24% ± SD 15%. Patient characteristics are presented in Table 1.

**Table 1:** Patient characteristics

Patient characteristic	All infants (n = 92)	Missing
Female	77 (84)	0 (0)
Age at first US (in months)	3.4 ± 1.2	0 (0)
US of left hip	65 (71)	0 (0)
Alpha angle		
Left hip	39 ± 5.7	25 (38)
Right hip	41 ± 7.0	11 (41)
Age at 5 years follow-up radiograph	5.0 ± 1.0	3 (3)
Graf D	10 (11)	0 (0)
FHC-index (%)	24 ± 15	0 (0)

*Categorical data is noted as ‘number (%)’, continuous data as ‘mean* ± *standard deviation’*

**Fig. 3:**
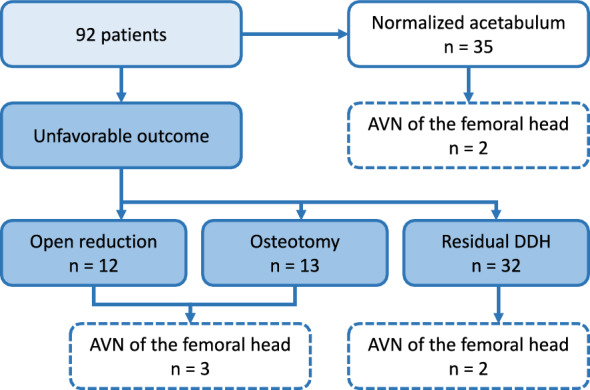
Lowchart of outcomes at final 5-year follow-up

At the five-year follow-up, 35 patients (38%) had no surgery and a normalized acetabulum. The remaining 57 patients (62%) had unfavorable outcomes: 12 required open reduction, 13 underwent pelvic osteotomy, and 32 had residual developmental dysplasia of the hip (DDH). All pelvic osteotomies were performed following the same ‘Pemberton’ surgical procedure as described by Huang et al. with an equal postoperative rehabilitation scheme of four weeks of hip spina casting of the involved hip. AVN was observed in three patients who had surgery and two with residual DDH. Detailed outcomes are shown in Fig. 3.

### Statistical shape model

**Fig. 4:**
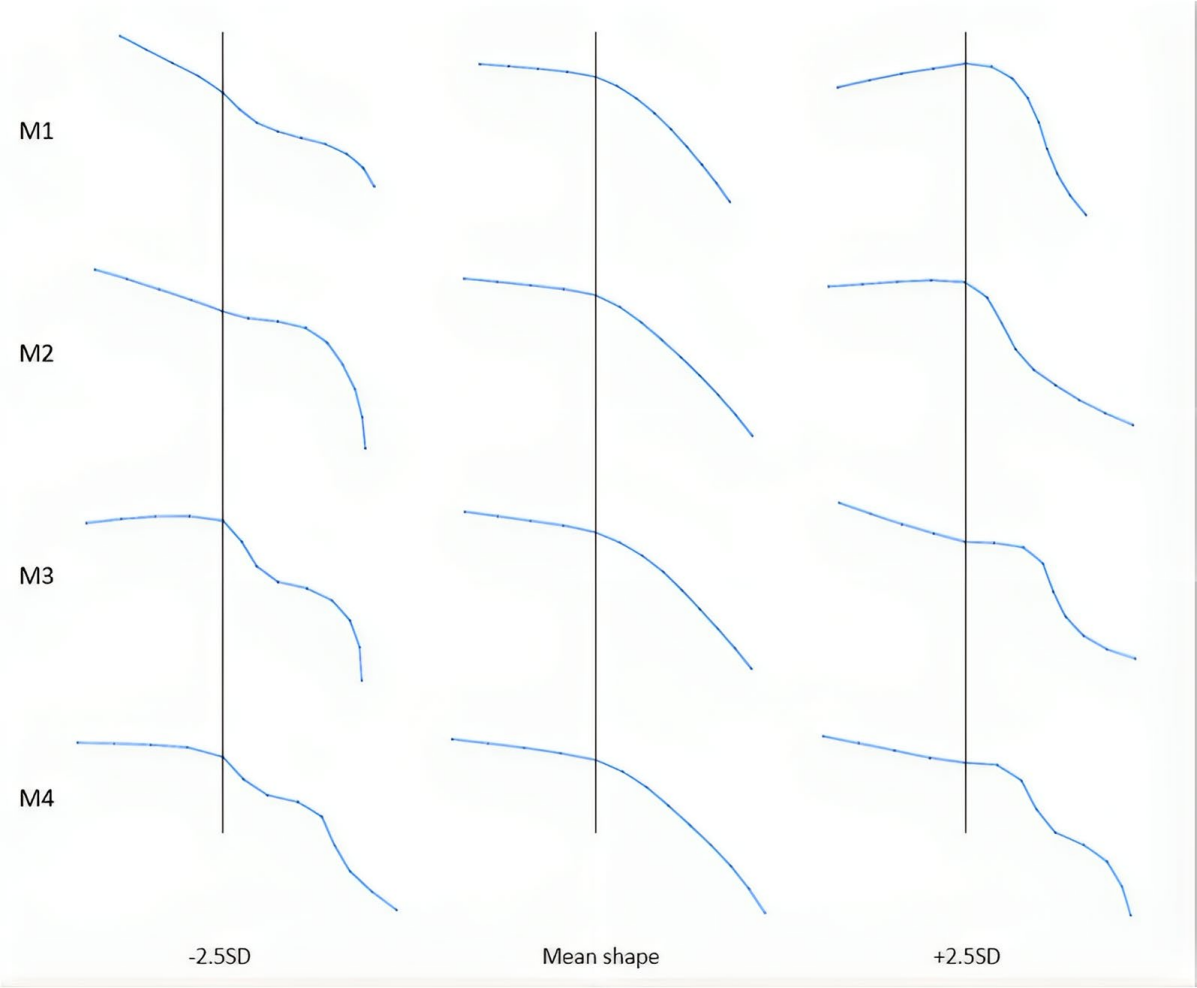
First four shape modes explaining 95% of shape variation. For clarity purposes, −2.5SD, 0SD, and + 2.5 SD from the mean shape are depicted for each mode. A vertical line is drawn through point 4 in all shapes, representing the start of the slope of the bony acetabular roof

The SSM identified four distinct shape modes, which explained 95% of shape variation. Mode 1 explained most of the shape variation (59%). The other three modes explained 37% of the shape variation: 23% by mode 2, 10% by mode 3, and 3% by mode 4. The different shape modes are depicted in Fig. 4.

### Shape modes and association with outcomes

Table 2 presents all odds ratios for continuous variables. Cut-off values for categories of both Z-scores of shape modes and FHC are shown in Table 3. Odds ratios for categorized variables are shown in Table 4.

**Table 2:** Odds ratios of association between modes and outcome: continuous variables

Mode	Odds ratio	P-value	Odds ratio	P-value
	Univariable analysis		Multivariable analysis	
Open reduction (first adverse outcome)				
1	0.75 [0.40, 1.41]	0.372	0.76 [0.41, 1.41]	0.386
2	1.13 [0.62, 2.06]	0.697	1.13 [0.62, 2.07]	0.690
3	1.40 [0.74, 2.66]	0.298	1.37 [0.73, 2.54]	0.326
4	0.82 [0.45, 1.49]	0.509	0.84 [0.45, 1.58]	0.594
Surgical intervention—open repositioning or osteotomy (second adverse outcome)				
1	0.56 (0.33, 0.96)	0.034*	0.56 (0.33, 0.95)	0.030**
2	1.25 [0.79, 1.98]	0.344	1.24 [0.76, 2.01]	0.388
3	1.36 [0.84, 2.20]	0.212	1.43 [0.87, 2.37]	0.160
4	1.12 [0.70, 1.79]	0.627	1.20 [0.73, 1.97]	0.476
Osteotomy or residual dysplasia with open repositions excluded (Third adverse outcome)				
1	1.17 [0.73, 1.88]	0.508	1.18 [0.71, 1.95]	0.520
2	1.00 [0.65, 1.56]	0.984	0.97 [0.61, 1.53]	0.888
3	0.86 [0.55, 1.33]	0.500	0.90 [0.56, 1.46]	0.670
4	2.00 [1.21, 3.38]	0.007*	1.99 [1.19, 3.34]	0.009**
Unfavorable outcome—open repositioning, osteotomy, or residual dysplasia- (fourth adverse outcome)				
1	1.11 [0.72, 1.71]	0.629	1.07 [0.69, 1.67]	0.765
2	1.03 [0.67, 1.59]	0.880	1.00 [0.64, 1.56]	0.999
3	1.04 [0.68, 1.60]	0.845	1.09 [0.70, 1.70]	0.699
4	1.80 [1.12, 2.90]	0.015*	1.80 [1.12, 2.91]	0.016**

Noted as OR [95% CI]. *Statistically significant values. ** Omnibus test > 0.05, complete model insignificant

**Table 3:** Values for categorized independent variables

**Mode**	Q0 (n = 18)	Q1 (n = 19)	Q2 (n = 18)	Q3 (n = 19)	Q4 (n = 18)
**M1**	< − 0.610	− 0.610–0.291	− 0.291–0.077	0.104–0.855	> 0.855
**M2**	< − 0.846	− 0.846–0.419	− 0.419–0.214	0.214–0.813	> 0.813
**M3**	< − 0.688	− 0.688–− 0.280	− 0.280–0.341	0.341–0.773	> 0.773
**M4**	< − 0.799	− 0.799—− 0.057	− 0.057–0.292	0.292–0.794	> 0.794
	Category 0 (n = 3)	Category 1 (n = 21)	Category 2 (n = 68)		
**FHC**	> 58%	33–58%	< 33%		

Q: quintile, equal groups containing 20% of patients. Numbers displayed are standard deviation, unless otherwise noted

**Table 4:** Odds ratios of association between modes and outcome: categorized variables

Mode	Q1	P-value	Q2	P-value	Q3	P-value	Q4	P-value
Open reduction (first adverse outcome)								
1	0.31 [0.05, 1.84]	0.195	0.00 [0.00,-]	0.998	0.14 [0.02, 1.39]	1	0.31 [0.05, 1.84]	0.195
2	2.13 [0.34, 13.4]	0.419	1.00 [0.13, 8.00]	1.00	0.00 [0.00,-]	2	2.13 [0.34, 13.4]	0.419
3	0.19 [0.02, 1.94]	0.163	0.21 [0.02, 2.06]	0.179	0.00 [0.00,-]	3	0.19 [0.02, 1.94]	0.163
4	2.86 [0.48, 17.1]	0.250	1.00 [0.13, 8.00]	1.00	0.94 [0.12, 7.50]	4	2.86 [0.48, 17.1]	0.250
Surgical intervention—open repositioning or osteotomy (second adverse outcome)								
1	0.58 [0.15, 2.21]	0.422	0.48 [0.12, 1.93]	0.301	0.07 [0.01, 0.64]	**0.018**	0.48 [0.12, 1.93]	0.301
2	2.55 [0.61, 10.7]	0.202	1.00 [0.21, 4.81]	1.00	0.41 [0.07, 2.59]	0.344	2.23 [0.52, 9.59]	0.283
3	0.53 [0.12, 2.33]	0.404	0.77 [0.19, 3.19]	0.718	0.24 [0.04, 1.37]	0.108	1.60 [0.41, 6.18]	0.495
4	1.20 [0.29, 4.94]	0.800	0.74 [0.16, 3.38]	0.701	1.20 [0.29, 4.94]	0.800	0.74 [0.16, 3.38]	0.701
Osteotomy or residual dysplasia with open repositions excluded (Third adverse outcome)								
1	0.78 [0.19, 3.13]	0.723	0.61 [0.15, 2.49]	0.493	0.79 [0.20, 3.06]	0.730	1.46 [0.35, 6.11]	0.606
2	1.63 [0.40, 6.63]	0.493	1.29 [0.32, 5.17]	0.723	1.03 [0.27, 3.99]	0.968	0.89 [0.22, 3.66]	0.870
3	0.56 [0.13, 2.36]	0.432	0.50 [0.12, 2.06]	0.337	0.29 [0.07, 1.21]	0.089	0.44 [0.10, 1.92]	0.273
4	1.71 [0.40, 7.27]	0.467	1.96 [0.47, 8.11]	0.356	3.46 [0.84, 14.3]	0.087	4.03 [0.95, 17.2]	0.060
Unfavorable outcome—open repositioning, osteotomy, or residual dysplasia- (fourth adverse outcome)								
1	0.69 [0.18, 2.62]	0.583	0.63 [0.16, 2.41]	0.495	1.08 [0.27, 4.29]	0.909	1.30 [0.31, 5.39]	0.718
2	0.86 [0.22, 3.32]	0.823	1.30 [0.31, 5.39]	0.718	0.56 [0.15, 2.10]	0.387	1.00 [0.25, 4.00]	1.00
3	1.08 [0.25, 4.60]	0.920	0.39 [0.10, 1.54]	0.176	0.35 [0.09, 1.36]	0.129	1.35 [0.30, 6.13]	0.701
4	1.71 [0.46, 6.37]	0.421	2.00 [0.52, 7.69]	0.313	2.17 [0.57, 8.26]	0.257	2.60 [0.65, 10.4]	0.176

Q: quintile, equal groups containing 20% of patients. Q0 is reference category. Noted as OR [95% CI]

Bold value highlights the significant association

No statistically significant associations were found between the shape modes and the 12 dysplastic hips (13%) needing open reduction. However, a statistically significant association was found between positive values of Mode 4 and an unfavorable outcome at age 5 (fourth adverse outcome), with an OR for univariable and multivariable regression analysis of respectively 1.80 [95% CI 1.12, 2.90] (p = 0.015) and 1.80 [95% CI 1.12, 2.91] (p = 0.016), indicating that a + 1 SD deviation in Mode 4 was linked to an unfavorable outcome. Additionally, positive values of Mode 4 were significantly associated with the third adverse outcome (a pelvic osteotomy or residual DDH at age 5), with an OR of 1.90 [95% CI 1.18, 3.13] (p = 0.009) and 1.90 [95% CI 1.15, 3.05] (p = 0.012), respectively. The logistic regression model did not reveal a clear pattern for the ORs of the categorized shaped modes.

Negative Mode 1 values showed a statistically significant association with all patients who had an open reduction or an osteotomy before age 5 (second adverse outcome), presenting odds ratios of 0.56 [95% CI 0.33, 0.96] (p = 0.034) and 0.56 [95% CI 0.33, 0.95] (p = 0.030), respectively. This indicates that a − 1 SD deviation in Mode 1 was linked to a surgical procedure.

FHC was not a significant predictor in the association between modes and outcome, suggesting no significant association was found between the extent of undercoverage of the femoral head and outcome. No significant associations between FHC and modes and outcomes were found for hips defined as type Graf D, nor for the secondary outcome signs of a history of AVN (data not shown).

### Interobserver reliability

The weighted (in terms of variation explained) overall ICC was 0.82 [95% CI 0.71, 0.88).

## Discussion

This study aimed to improve the prediction of the clinical course and outcome at age five of decentered hips (Graf D, III an IV), as diagnosed on the first US made in the first months after birth, by identifying acetabular shape variants on these US images using a statistical shape model (SSM). More specifically, the identified shape variants were studied for the prediction of the likelihood of open reduction or insufficient hip development (osteotomy or residual DDH).

Ultrasound images of 92 neonatal hips were included to construct a statistical shape model. No baseline characteristics nor currently used ultrasound classifications, such as the Graf classification and FHC, were found to be associated with outcomes. In this cohort, four shape modes explained 95% of the shape variation. The principal findings of this study revealed a significant association between two of the identified shape modes and the risk for open reduction, the need for an osteotomy, or residual DDH at age 5 (4th adverse outcome). More specifically, positive values of Mode 4 were significantly correlated with open reduction, an osteotomy, or residual DDH at age 5 (4th adverse outcome), but also with the risk for an osteotomy or residual DDH when hips with open reduction were excluded (3rd adverse outcome). This indicates that Mode 4, derived from the initial ultrasound images of decentered hips (Graf D, 3–4) with SSM, might predict an increased risk for an unfavorable course. Furthermore, positive values in Mode 1 were associated with hips that had either open reduction or an osteotomy before age 5 (2nd adverse outcome). Mode 1 was, however, not statistically significantly associated with the hips that had either an osteotomy or residual DDH at age 5 (3rd adverse outcome). Although the indication for surgery could be considered arbitrary, Mode 1's strong correlation with surgical intervention, as opposed to its lack of correlation with the 3rd adverse outcome, could indicate its potential to differentiate between mild and more severe dysplastic development.

In line with the study of Bonsel et al. for centered stable dysplastic hips, the results from this study show that the use of SSM to quantify and analyze the acetabular shape of infantile dysplastic hips on the initial ultrasound images seems to be associated with a better prediction on outcomes at follow-up. (2022b) Specific acetabular shape modes were found and associated with different outcomes at follow-up. These associations cannot be found in the current classification systems, such as the Graf or FHC classifications. The findings of this study might be improved with validation in larger and more heterogeneous cohorts to make a prognostic imaging model for initial neonatal decentered DDH. This model should be used for more specific detection of at-risk patients to improve treatment strategies, including tailored interventions or cost-effective monitoring approaches.

The literature indicates the need for improving tools for prognosis and treatment strategies of decentered DDH. Sanker et al. found a 9% failure rate of closed reductions in 78 decentered hips, while Morris et al. reported a 47% need for secondary surgical intervention after initial successful closed reduction in 104 patients. However, no prognostic tools exist that indicate which decentered hips are at risk for unfavorable hip development and should be monitored more intensively. [16, 19]

The following limitations of this study must be taken into account. The study was conducted with a small cohort in a single center and a variation in the timing of the initial ultrasound and final X-ray. Partially due to the retrospective design, missed traumas and (not-recognized genetic) comorbidities could have influenced outcomes. Due to the limited sample size, sub-analysis and cross-validation were considered unreliable. No adjustment for multiple testing was made since this study was a hypothesis-generating study instead of a (hypothesis) confirming study. Although the risk of false positives is regarded as low in this multiple-hypothesis study, this risk should be of concern and statistically taken care of in future studies. This study only used a 2D analysis of bony aspects in a 3D anatomical disorder. However, the intention was to use the current techniques to make a US image of the hip for diagnosing DDH and to have a method that can easily be implemented in the current practices. The interobserver reliability analysis demonstrated overall feasibility and reproducibility, although the individual ICCs of Mode 2–4 were < 0.70. However, one should consider the progressively smaller share in the total variance of these Modes in combination with the small sample size. A larger sample size, more raters, or automatic instead of manual placement of points will probably lead to improved reliability.

For future research, a more extensive, more heterogeneous multicenter validation study should be conducted to deliver data for a more thorough analysis in—for example—subgroups and validation of results through multiple testing. The SSM model might also be optimized with more or adjusted data points per US image, potentially leading to more refined shape patterns among the separate modes and more specific prognostic methods by categorization of the SD.

## Conclusion

Using SSM to quantify and analyze acetabular shapes on US images of untreated decentered dysplastic hips (Graf D, III, IV) might lead to a better prediction of outcomes at follow-up. In this single-center population, it was possible to categorize the acetabular shape on the initial US image into four different modes, explaining 95% of the shape variations, of which two shape modes had a statistically significant association with well-defined outcomes within the first five years of life. These findings might help develop a better, more prognostic classification of DDH. Of the four shape modes, Mode 4 was associated with a defined unfavorable outcome at age 5. Mode 1 was significantly associated with open reduction or an osteotomy, which might indicate that hips with this shape morphology are considered more severe cases of DDH. In more extensive multi-center studies, validating specific shape characteristics on US images might further improve the prognostic value for specific outcomes.

## Data Availability

The datasets used and/or analysed during the current study are available from the corresponding author on reasonable request.
